# The neural representation of abstract words may arise through grounding word meaning in language itself

**DOI:** 10.1002/hbm.25593

**Published:** 2021-07-15

**Authors:** Annika Hultén, Marijn van Vliet, Sasa Kivisaari, Lotta Lammi, Tiina Lindh‐Knuutila, Ali Faisal, Riitta Salmelin

**Affiliations:** ^1^ Department of Neuroscience and Biomedical Engineering Aalto University Aalto; ^2^ Aalto NeuroImaging Aalto University Aalto

**Keywords:** abstract concepts, concrete words, decoding, machine learning, MEG, RSA, semantics, word processing

## Abstract

In order to describe how humans represent meaning in the brain, one must be able to account for not just concrete words but, critically, also abstract words, which lack a physical referent. Hebbian formalism and optimization are basic principles of brain function, and they provide an appealing approach for modeling word meanings based on word co‐occurrences. We provide proof of concept that a statistical model of the semantic space can account for neural representations of both concrete and abstract words, using MEG. Here, we built a statistical model using word embeddings extracted from a text corpus. This statistical model was used to train a machine learning algorithm to successfully decode the MEG signals evoked by written words. In the model, word abstractness emerged from the statistical regularities of the language environment. Representational similarity analysis further showed that this salient property of the model co‐varies, at 280–420 ms after visual word presentation, with activity in regions that have been previously linked with processing of abstract words, namely the left‐hemisphere frontal, anterior temporal and superior parietal cortex. In light of these results, we propose that the neural encoding of word meanings can arise through statistical regularities, that is, through grounding in language itself.

## Graphical abstract

A statistical model using word embeddings extracted from a text corpus was used to train a machine learning algorithm to successfully decode the MEG signals evoked by written words. In the model, word abstractness emerged from the statistical regularities of the language environment. The study provides proof of concept that a statistical model of the semantic space can account for neural representations of both concrete and abstract words.

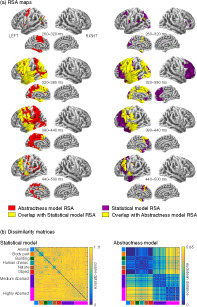

## 1 INTRODUCTION

Understanding abstract and concrete concepts is a fundamental aspect of human language that enables us to discuss matters ranging from everyday objects to fantastic stories of fiction. A common view is that word meanings are grounded in experiences with the world (Binder et al., 2016; Kiefer & Pulvermüller, 2012; Martin, 2007; Vigliocco & Vinson, 2007). For example, the word “tomato” is linked with the look, feel and taste of a tomato. This view of lexical semantics asserts that these types of physical associations form the building blocks of how words are encoded in the brain. However, the grounding framework fails to account for abstract words, which lack physical referents and, in many cases, an emotion or an internal state to which the word meaning can be grounded. This issue can be overcome if word meanings can also be grounded in the experience of language. That is, if language is seen as another physical environment that a person can interact with, language becomes equivalent to perceptual data, enabling what has been coined as linguistic scaffolding (Clark, 2006). In line with this conceptualization, the representations of both concrete and abstract words will mirror, not merely physical regularities, but also regularities in the language environment. Here, we propose that modern multivariate methods applied on time‐sensitive MEG signals may serve as useful tools for investigating this issue. We aim to demonstrate that emergent properties based on the statistical regularities of our language environment capture the abstractness/concreteness of a word, and that these properties are correlated with how words are represented in the brain, as reflected in the neural signals elicited during word reading.

Computational models in the field of natural language processing (NLP) have demonstrated that a distributed representation of word meanings can be derived from the context in which the words are used. The core idea of these models is to find an optimal decomposition of semantics that can represent each unique concept without excessive use of memory or processing effort. Statistical regularities in the training data (typically a large text corpus) will drive the organization of the distributed representations, which together form a semantic space. Categorical structures, such as that of abstract and concrete words, can emerge in such a semantic space (Hollis & Westbury, 2016). These models rely on the same general computational principles that underlie brain function, namely Hebbian learning (Hebb, 1949) and basic principles of optimization (Friston, 2012; Zipf, 1949). If we further assume that a large text corpus is a fair estimate of the natural language environment that our brains are immersed in, a statistical model of a text corpus could serve as a reasonable approximation of the organizational principles of word meanings also at the level of the brain. This assumption builds on the recognition that intralinguistic distributional and sensorimotor data are interdependent (Andrews, Frank, & Vigliocco, 2014) and allows for a model to approximate the meaning of both concrete and abstract words by the same general computational principles. Indeed, unsupervised methods that build a distributed semantic space through optimization have been shown to find a dimension of conceptual concreteness regardless of whether the model is trained on a text corpus (Hollis & Westbury, 2016) or image data (Kiela, Hill, Korhonen, & Clark, 2014).

Here, we built a statistical model of word meanings by applying the word2vec algorithm to a large text corpus of the Finnish internet. The algorithm was developed in the field of natural language processing (Mikolov, Chen, Corrado, & Dean, 2013), and it bases its notion of semantic similarity on the principle that two words are similar if they occur within a similar linguistic context, even if they never directly co‐occur. Word2vec will discover thematic relationships (bear – zoo), that is, concepts that either serve complementary roles or that co‐occur in common situations, locations and/or times, but do not necessarily share perceptual or functional characteristics (De Deyne, Verheyen, & Storms, 2016; Lin & Murphy, 2001).

Systematic patterns in the (language) environment can give rise to qualitative differences in the way concrete and abstract words are represented or processed, even if those word types share the same organizational principles. Behaviorally, concrete words elicit faster reaction times than abstract words (James, 1975). Patient data suggest a double dissociation between abstract and concrete word types as either one may be selectively impaired (Reilly, Peelle, & Grossman, 2007; Warrington, 1975). Furthermore, numerous neuroimaging studies have shown that processing of abstract and concrete words activate brain areas differently (for a meta‐analysis see Wang, Conder, Blitzer, & Shinkareva, 2010). Generally, processing of abstract words (nouns in particular) activates classical language areas, such as the inferior frontal gyrus and the middle/superior temporal gyrus, more strongly than processing of concrete words. In contrast, concrete words seem to activate the posterior cingulate, precuneus, fusiform gyrus, and parahippocampal gyrus more strongly than abstract words (Wang et al., 2010). Electrophysiological evidence reports a stronger and longer‐lasting neural response for concrete than abstract words at around 400 ms after word onset (Huang, Lee, & Federmeier, 2010).

Important advances regarding the structure of the semantic space in the brain have been made by using multivariate analyses and decoding (Huth, de Heer, Griffiths, Theunissen, & Gallant, 2016; Mitchell et al., 2008; Pereira et al., 2018). However, these studies have not addressed the *how*, *where*, and *when* abstract words are represented in the brain. By linking brain activity during word reading, measured by magnetoencephalography (MEG), with a statistical model of semantics, we can tap into both the time and location where semantic information is processed. This entails overcoming the difficulty of decoding written stimuli from electrophysiological signals; a previous EEG study found that the average categorical classification accuracy for pictures was 77% and above chance for all participants, whereas decoding concrete written words in the same participants was only successful in two participants, maximally scoring 68% correct (Simanova, Hagoort, Oostenveld, & van Gerven, 2014).

A statistical model of semantics is theoretically appealing as it contains a neurally feasible way to describe how semantic representations may arise in the brain. However, a statistical model does not in itself contain any information about how the emergent dimensions of the semantic space should be interpreted. To aid us with this, we created a behaviorally derived classification of the level of abstractness of a word, which we call the Abstractness model. By comparing whether the Statistical and the Abstractness models capture the same information, we are able to interpret whether some part of the statistically derived semantic space relates to word abstractness. We can then test whether any shared information is also mirrored in the neural signals elicited during word reading. To do this, we test if a supervised machine‐learning method can successfully model the relationship between the MEG data for each stimulus word and the corresponding feature decomposition of the word from the Statistical model. As one step further, we use representational similarity analysis (RSA, Kriegeskorte, Mur, & Bandettini, 2008) to discover time bins and cortical regions where the variation in the source estimate of the MEG signal is similar to the variation in the Statistical model of word meanings.

## 2 MATERIALS AND METHODS

### 2.1 Participants

MEG measurements were performed on 20 volunteers (mean age 21 years, sd 3.6, range 18–34; 50% identified themselves as females). All participants were native Finnish speakers, had normal or corrected to normal vision, and were scored as highly right‐handed on the Edinburgh handedness questionnaire. All participants were healthy, reported no diagnosed neurological disorders or reading disabilities and were compensated financially for their participation. Informed consent was obtained from all participants.

In addition, a total of 408 respondents filled behavioral questionnaires, created either for stimulus evaluation or to collect the behavioral feature sets (see more information below). The respondents were volunteers who were reimbursed for the effort with movie tickets. All respondents had Finnish as their first language, their mean age was 27 years (sd 7, range 19–63) and 65% identified themselves as females.

The study was approved by the Aalto University Research Ethics Committee in agreement with the Declaration of Helsinki.

### 2.2 Stimuli

The stimuli consisted of 118 nouns grouped into two main categories: concrete (59 words) and abstract (59 words). The two main categories did not differ significantly in lemma frequency (unpaired two‐tailed *t* test: *t*(58) = −1.1, *p* = .28), based on the prevalence in a large corpus of internet sites in Finnish (1.5 billion words). All words were within the 90th percentile of the corpus distribution and can thus be considered common, high frequent words. The length of the stimulus words ranged from 3 to 10 letters and did not differ between the abstract and concrete words (*t*(58) = − 1.9, *p* = .065).

All stimulus words were assessed on a scale from 1 to 7 on the level of concreteness, estimated age of acquisition (AoA), imageability, concreteness, emotionality and valence, in a web‐based behavioral questionnaire. The assessment was done by 13 naïve respondents that did not partake in any other part of the present study. The concrete words were judged as very concrete (mean rating: 6.5 [sd 0.5]). The abstract category contained 30 highly abstract words (mean concreteness: 2.0 [sd 0.9], mean imageability: 2.3 [sd 1.0]) and 29 medium‐abstract words (mean concreteness: 3.9 [sd 0.7]; mean imageability: 4.1 [sd 0.8]). It has previously been shown that highly imaginable words tend to be acquired earlier than words with low imageability (Stadthagen‐Gonzalez & Davis, 2006). Also in the present stimulus set the estimated AoA for concrete words (mean rating 1.2 [sd 0.3]) was significantly lower (*t*(58) = −9.2, *p* < .001) as compared to abstract words (mean rating 2.1 [sd 0.6]). There was no difference in valence between the word categories (*t*(58) = 1.20, *p* = 9.23).

The concrete words were sub‐grouped according to the categories that have been derived from specific impairments following brain damage (Caramazza & Shelton, 1998; Sartori, Miozzo, & Job, 1993; Warrington & Shallice, 1984), namely Animal (e.g., dog, bear), Body part (e.g., hand, foot), Building (e.g., bridge, hospital), Human character (e.g., child, princess), Nature (e.g., island, fire), and Object (e.g., hammer, ball). Each category contained 10 items, with the exception of the Human character category that only contained 9 items. The full list of the stimuli is reported in the Table S1.

### 2.3 Corpus‐derived statistical model of semantics

The corpus‐derived Statistical model was created using a continuous skip‐gram word2vec algorithm (Mikolov, Chen, et al., 2013) which looks for co‐occurrences between a particular word and the neighboring words (i.e., linguistic context) and represents this information as a N‐dimensional vector. The model was trained on the same corpus that was used to estimate the frequency of the stimulus words, which contains a large sample of internet sites in Finnish (1.5 billion words) (Kanerva, Luotolahti, Laippala, & Ginter, 2014), using negative sampling which is a computationally efficient method to approximate the conditional log‐likelihood of the model (Mikolov, Sutskever, Chen, Corrado, & Dean, 2013). In the resulting vector space, words that share a similar linguistic context are located close to each other. Here, we used the default vector length of 300. A context window before and after the stimulus word was used to capture the co‐occurrences.

Different sizes for the context window were tested (3, 5, 10), with the size of 10 yielding best decoding performance (see Section 2.7) and hence selected to be carried throughout the entire analysis. The vectors corresponding to the words that were presented as stimuli were visualized using theUniform Manifold Approximation and Projection (UMAP) algorithm, with 15 neighbors and 500 iterations (McInnes, Healy, & Melville, 2018).

### 2.4 Experimental design

During the MEG recording written words were presented one by one in a black monospaced font (Courier New) on a gray background. Each word was presented for 150 ms followed by a blank screen for 950 ms. Between trials, a fixation cross was presented for 1,000 ms. Each word was presented a total of 20 times, over the course of two one‐hour long MEG sessions that took place on separate days. The sessions included breaks of a few minutes every 20 min. The order of the stimulus words was randomly determined for each day, so that each stimulus was repeated 10 times each day but words were never repeated back to back.

In order to ensure the compliance of the participants, 10% of the trials were followed by a catch trial, during which the end part of a sentence was presented on the screen and the subject was instructed to determine if the preceding word would make sense as the first word of this sentence. For example, the word “beauty” might be followed by the phrase “*… is in the eyes of the beholder*” in which case the correct answer would be “*yes*” as the phrase “*beauty is in the eyes of the beholder*” is a reasonable sentence. Stimuli were presented using the Presentation software by Neurobehavioral Systems.

### 2.5 MEG and MR measurements

MEG was measured using a whole‐head Vectorview MEG device (Elekta Oy, Helsinki, Finland) with 102 triplet sensor elements, each containing two planar gradiometers and one magnetometer. The data was filtered at 0.003–200 Hz and sampled at 1,000 Hz. Eye movements and blinks were recorded using an electro‐oculogram (EOG), configured as pairs of electrodes placed vertically and horizontally around the eyes. The head position with respect to the scanner was determined by four indicator coils placed on the forehead and behind the ears. The head position was measured at the beginning of each 20 min segment of the recording session. The position of the coils, as well as approx. 60 additional points along the surface of the head, were determined in a coordinate system spanned by three anatomical landmarks (the left and right preauricular points and the nasion) using a 3D Polhemus digitizer (Polhemus, Colchester, VT). The MEG data was co‐registered to the anatomical MR images based on the anatomical landmarks and the additional data points, using the Elekta Maxfilter software package.

Anatomical MR images were scanned on a separate day using a 3T MAGNETOM Skyra scanner (Siemens Healthcare, Erlangen, Germany), a standard 20‐channel head–neck coil and a T1‐weighted MP‐RAGE sequence.

### 2.6 MEG data analysis

The MEG data was preprocessed by aligning head positions from the different data segments and different days into one head position and removing external noise sources using the spatiotemporal signal space separation method (Taulu & Simola, 2006) in the Elekta Maxfilter software package. Artefactual signals due to eye blinks were suppressed using a PCA approach (Uusitalo & Ilmoniemi, 1997), where the 1–2 components that capture the most variance of the average MEG response to blinks were removed from the raw data.

Event‐related epochs were extracted from the gradiometer data from 200 ms before to 1,000 ms after each word onset and averaged across the multiple presentations of the same item. Since we are mostly interested in cortical signals, we opted to only use the gradiometers for the multivariate analyses, as they have a slight edge in signal‐to‐noise ratio over the magnetometers for superficial sources. The event‐related responses were baseline‐corrected to the interval from −200 ms until the word onset and low‐pass filtered at 25 Hz. Any trials where the signal exceeded 3,000 fT/cm were removed (max. 1 trial per word).

Separate source‐level estimates for each stimulus item, averaged across the 20 repetitions of this item, were computed using Minimum Norm Estimates (MNE) (Gramfort et al., 2013; Gramfort et al., 2014; Hämäläinen & Ilmoniemi, 1994) constrained to the cortical surface. The volume conduction model was based on the individual structural MRIs using the Freesurfer software package (Dale, Fischl, & Sereno, 1999; Fischl, Liu, & Dale, 2001) and modeled as a single‐compartment boundary element model with an icosahedron mesh of 2,562 vertices in each hemisphere for each participant.

In the inverse solution, currents perpendicular to the cortical surface were favored by setting the loose orientation constraint parameter to 0.3, and depth‐weighting was used to reduce the bias towards superficial sources (Dale et al., 2000). The source estimate regularization parameter lambda was set to 0.1. An empirical noise‐covariance matrix based on the baseline period across all stimuli was used for noise normalizing of the source estimates, resulting in dynamical statistical parametric maps (dSPM; Dale et al., 2000). Lastly, the individual source estimates were morphed onto FreeSurfer's average template brain.

### 2.7 Zero‐shot decoding

In order to determine whether the Statistical model of the semantic space is a good description of the neural responses during word reading, we used linear ridge regression to learn a linear mapping between the sensor‐level MEG evoked responses and the Statistical model (Pedregosa et al., 2011).

To reduce the dimensionality of the input data, the MEG responses were downsampled by creating 20‐ms bins within the time window 0–800 ms relative to the onset of the stimulus presentation, resulting in 40 bins. To evaluate the performance of the model over time, we applied the model across the bins in a sliding window fashion, using a window size of 5 bins. For each of the 118 stimulus words, the averaged signals for each bin at each of the 204 sensor locations were concatenated into a single vector, yielding a 118 × 1,020 input matrix. The target matrix contained the word2vec vector for each of the 118 stimulus words, yielding a 118 × 300 matrix. The columns of both the input and target matrices were *z*‐transformed before being entered into the ridge regression.

The resulting mapping was evaluated by attempting to match two previously unseen segments of MEG data with two unseen stimulus words. This is referred to as zero‐shot decoding (Palatucci, Pomerleau, Hinton, & Mitchell, 2009). To do this, the zero‐shot approach employs two steps. First, the algorithm uses the learned mapping between the MEG data and the individual features to translate the two MEG segments into two predicted feature vectors. The identity of the two unseen stimulus words is then determined by comparing the cosine distance between the predicted vectors and the original Statistical model vectors for these items (Sudre et al., 2012). This binary discrimination task is carried out for all possible pairs of two stimulus words, using the remaining 116 words for training. For each participant, we report the mean accuracy across all word pairs, which ranges between 50% (i.e., algorithm fails to distinguish between words) and 100% (i.e., successful discrimination between all stimulus words).

To test whether the obtained accuracy scores were significantly higher than chance level, the zero‐shot classification procedure was repeated 1,000 times on randomly permuted data. Random data was produced by choosing the data of one subject at random and randomizing the assignment between the word labels and the MEG data segments. As *p*‐value, we report the percentage of accuracy scores for the random permutations that equaled or exceeded the accuracy score obtained on real data.

### 2.8 RSA analysis

RSA (Kriegeskorte et al., 2008) was performed between the source localized MEG data and Statistical model, using the MNE‐RSA software package (https://github.com/wmvanvliet/mne-rsa). For the Statistical model, a single word‐to‐word dissimilarity matrix (DSM) was created by computing the Pearson correlation *r* across the feature vectors for each possible word pair, and using 1 − *r* as the dissimilarity score. The values along the diagonal (the dissimilarity between a word and itself) were set to zero.

The MEG data underwent the same downsampling and *z*‐transformation procedure used for the zero‐shot learning. Then, for each subject, time bin and source‐level vertex, a word‐to‐word DSM was formed using a searchlight approach: the signal at all vertices within a certain radius of the vertex under consideration was assembled into a vector. To compute a reasonable DSM, enough signal variation inside a single searchlight patch is needed. Given the spatial smoothness of the MNE source estimate, utilizing data from a rather large patch of the cortex is motivated, and hence the radius of the searchlight patches was set to 2 cm. Pairwise comparisons were then carried out between all resulting vectors that represent the words using Pearson correlation, with 1 − *r* as dissimilarity score.

The RSA maps for each subject and each feature set were obtained by comparing the MEG‐based DSMs with the feature‐set DSMs using Spearman rank correlation. Finally, the RSA maps were analyzed across subjects using a cluster permutation test (Maris & Oostenveld, 2007) with a cluster threshold of *p* = .01 (one‐sample *t* test) and a cluster‐wide significance threshold set to *p* = .05. To create a random distribution of the data, 5,000 permutations were performed using random sign flips. Any clusters with a corresponding cluster *t*‐value that was lower than 95% of the randomly obtained cluster *t*‐values were pruned from the RSA maps. The remaining clusters were deemed significant (*p* ≤ .05).

To aid the interpretation of the main RSA, an additional RSA was calculated between the MEG data and a separate, questionnaire‐based model quantifying only the abstract – concrete dimension (Abstractness model; see below). This additional RSA was computed in the same manner as the main RSA between the MEG data and the Statistical model, with the exception that the Euclidean distance was used as the distance metric in the word‐to‐word DSM of the one‐dimensional Abstractness model.

### 2.9 Abstractness model

The Abstractness model was derived from a behavioral web‐based questionnaire answered by 10 naïve respondents (who did not respond to the stimulus assessment questionnaire). The respondents were asked to assess how well each of the 118 stimulus words could be classified as belonging to each of the predefined categories (Animal, Body part, Building, Human character, Nature (excluding animals), Object, Abstract words) using a scale from 1 to 7 (1 = does not belong to this category, 7 = a typical example to this category). As an example, for the word “problem” 100% of the respondents indicated that it was a typical example of the category Abstract word. However, for the remaining categories the agreement (“the word ‘problem’ does not belong to this category”) was somewhat less consistent (80–100%). From this data set, we extracted the abstractness scale to be used as an Abstractness model.

## 3 RESULTS

**FIGURE 1:**
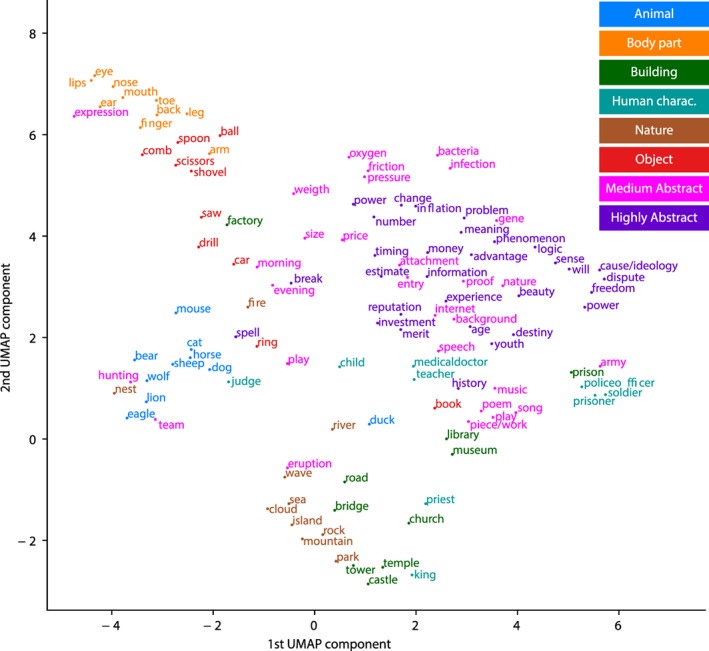
Visualization of the semantic space created by the Statistical model, obtained by projecting the word2vec vectors onto a two‐dimensional sheet using a Uniform Manifold Approximation and Projection for Dimension Reduction (UMAP). An interactive version of the figure is available at https://projector.tensorflow.org/?config=https://users.aalto.fi/~vanvlm1/redness1/projector_config.json

We used a Uniform Manifold Approximation and Projection for Dimension Reduction (UMAP) algorithm (McInnes et al., 2018) to visualize the internal structure of the high dimensional Statistical model (see Figure 1; for an interactive visualization see https://projector.tensorflow.org/?config=https://users.aalto.fi/~vanvlm1/redness1/projector_config.json (left‐hand panel options allow viewing by category, labels may be turned on from the top panel symbol A). The model spatially separates several of the categories. In particular, Body part, Nature and Animal, as well as the abstract words, all form distinct clusters; the Medium Abstract and Highly Abstract categories do not separate from each other. The abstract words (e.g., *problem*, *power*, *pressure*) group together and are distinct from the concrete words (e.g., *scissors*, *showel*, *sheep*).

**FIGURE 2:**
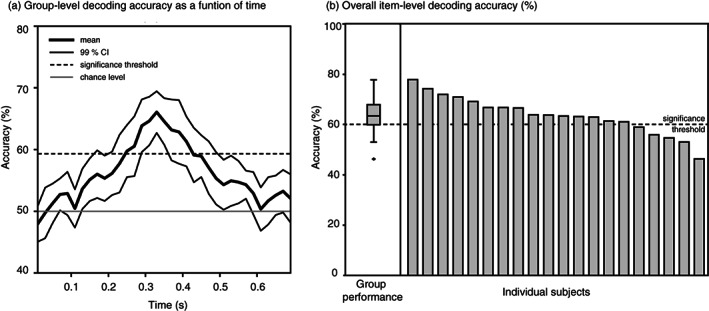
(a) Group‐level item‐level decoding accuracy as a function of time. (b) Overall item‐level decoding results. The box plot on the left shows the quartiles and the variation in the group performance (percent of successful decoding across all stimulus‐item pairs permutations). On the right are the individual scores of each participant. Accuracy scores above 59% and 60%, respectively, for the time‐resolved and the overall decoding results were deemed to be statistically significantly above the chance level based on a permutation test. CI, confidence interval

To determine whether the information in the Statistical model is mirrored in the brain activity during word reading, we used an item‐level decoding algorithm (Palatucci et al., 2009). The model was able to successfully discriminate between different stimulus words based on brain activation at 290–410 ms after the stimulus presentation (Figure 2a).

The decoding accuracy fell within the 95% confidence level for the majority of participants (Figure 2b). The adjusted chance level was determined statistically to be 60.1% (*p* < .05). The algorithm was thus able to find a mapping between the brain data and the Statistical model, which implies that the information encoded in the Statistical model is correlated with the information in the brain signal. The accuracy of the zero‐shot model was also evaluated using the cosine distance between the semantic location predicted from the MEG data and the semantic location indicated by the Statistical model (Figure S1). The time course of the cosine distance mimics the time course of the decoding accuracy (Figure 2a).

A breakdown of the item pairs used in the evaluation showed no clear between‐category advantage compared to within‐category comparisons, indicating that categorical structure is unlikely to be the sole driving factor of the decoding results (see Figure S2). However, by evaluating the decoding performance as a function of time using only the within‐category items of the abstract and concrete words, a slight advantage for the within‐concrete word decoding did emerge (see Figure S3). As the number of words in each category was half of the full set, comparing this result to that of the whole set results is difficult.

**FIGURE 3:**
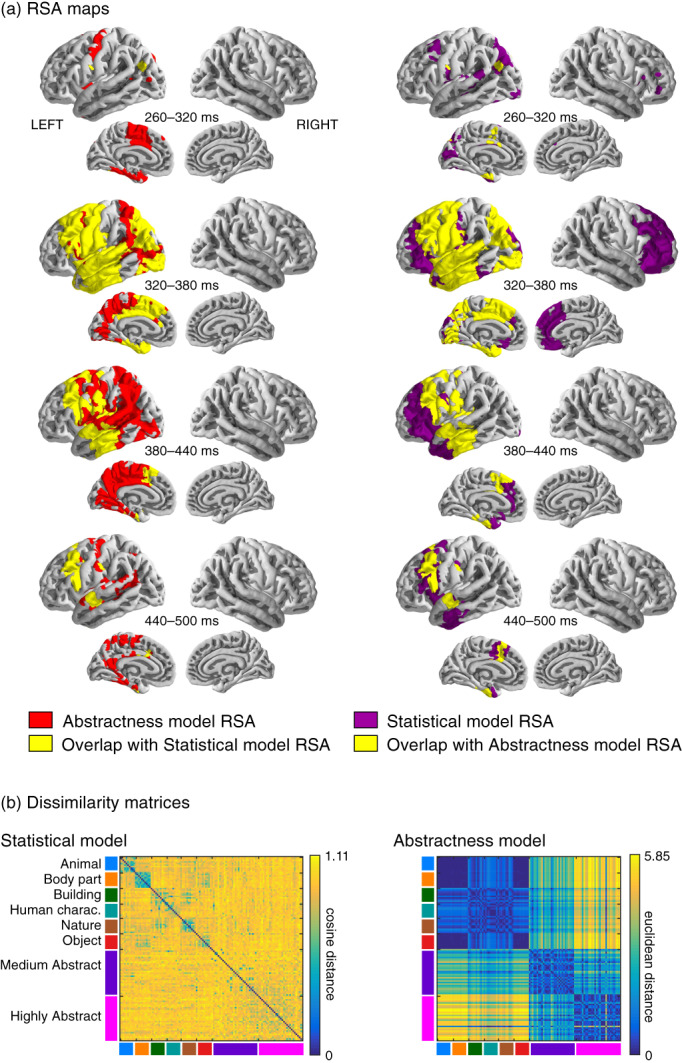
Comparison of the Statistical model and Abstractness model. (a) Representational similarity analysis (RSA) between the Statistical model and the MEG data (red) on the left and between the Abstractness model and the MEG data (purple) on the right. The overlap between the two RSAs is plotted in yellow. The results show all regions and time windows with statistically significant findings. For visualization purposes, the data was averaged over 60‐ms time windows. (b) Dissimilarity matrices of the Statistical model and the Abstractness model

We proceeded to investigate when and where the information expressed in the Statistical model is manifested in brain activation. Sensor‐level evoked responses (see Figure S4) and MNE‐dSPM source estimates (see Figure S5) show, on average, slightly more activation for abstract than concrete words in the temporal and inferior frontal regions. To investigate the relationship between the Statistical model and the brain activation further, we used an RSA (Kriegeskorte et al., 2008) between the MEG data and the semantic decompositions provided by the Statistical model (Figure 3a). Based on the UMAP (Figure 1), the dominant organizational principle of the Statistical model is the abstractness‐concreteness dimension. Therefore, to guide the interpretation of the RSA between the MEG data and the Statistical model (henceforth, Statistical model RSA), we additionally calculated a complementary RSA between the MEG data and a model used to quantify only the abstractness–concreteness dimension based on independently collected questionnaire data (see Methods; henceforth, Abstractness model RSA).

The Statistical model showed a high correlation with the Abstractness model (one‐dimensional questionnaire data) (Mantel test with Spearman's rho = 0.3, *p* < .001; 5,000 permutations used). This is probably due to the fact that both models clearly dissociate well between concrete and abstract words (see dissimilarity matrices in Figure 3b).

The spatio‐temporal overlap between the Statistical model RSA and Abstractness model RSA (Figure 3a) suggests that neural activity in the highlighted cortical regions contains information incorporated in both model types. A large portion of this information is related to the abstractness dimension, as shown by the high correlation between the Abstractness model and the Statistical model (see above).

The earliest neural response that was significantly correlated with the Statistical model was observed in the lateral superior posterior temporal cortex and medial occipital cortex at 270 ms. At 290–350 ms after stimulus presentation, the correlation advanced via the middle temporal, lateral and medial parts of the parietal and precentral cortices to the anterior temporal and frontal cortices. Activation in the temporal and frontal cortices was correlated with the Statistical model until 450 and 470 ms, respectively. Neural activity uniquely correlated with the Statistical model was also found in the right frontal cortices at 290–350 ms.

There was substantial overlap between the Statistical model RSA and the Abstractness model RSA in the precentral gyrus starting at 310 ms and extending into large portions of the superior and medial frontal cortex until 450 ms post stimulus presentation. The overlap occured in temporally distinct time windows in the superior frontal (320–370 ms) and the inferior frontal cortex (410–450 ms). Another prominent overlap was observed in the middle and anterior parts of the temporal cortex, including the ventro‐medial parts (320–430 ms). Overlap was also observed in the parietal cortex (starting from the angular gyrus) at 310–350 ms.

The areas highlighted uniquely by the Abstractness model RSA were found in the left precuneus (290–410 ms), the left inferior precentral gyrus (inferior parts 370–390 ms; superior and medial parts 370–410 ms), as well as the left posterior temporal cortex and temporo‐parietal junction (310–430 ms).

A complementary version of the RSA using partial correlation can be found in the supplementary information (see Figure S6).

## 4 DISCUSSION

The results of the present study show that a semantic space generated based on word co‐occurrence in sentential contexts has an emergent abstract and concrete categorical structure and can be used to describe the neural substrates of word meanings. The salient abstract‐concrete dimension of this corpus‐derived semantic space is in line with a previous observation that the semantic space generated by the word2vec algorithm is heavily loaded on word concreteness (Hollis & Westbury, 2016). The alignment of words rated as medium abstract between the concrete and abstract words in the semantic space further suggests that the abstract‐concrete distinction is, rather, a continuum. The categorical grouping within concrete words only partly aligned with the predefined taxonomic categories found in behavioral feature sets (e.g., Devereux, Tyler, Geertzen, & Randall, 2014). Behavioral and statistical models of semantics may thus be largely complementary descriptors of semantic organization. We found no clear categorical structure within the abstract words. However, this is to be expected given that the abstract stimuli did not follow a predefined categorical substructure, unlike the concrete stimulus words which were selected based on categories that have been linked to category‐specific impairments following brain damage.

The statistical properties of word meanings, derived through co‐occurrences in the text corpus, were successfully used to decode the identity of concrete and abstract written words based on their MEG responses, thereby showing that a statistical model of semantics serves to describe the organization of word meanings in the brain. The main correlations between brain activity and the Statistical model were found in the left precentral, frontal and temporal cortex as well as in superior and medial parietal areas. In the time domain, the decoding algorithm was most successful at decoding the word identity at 290–410 ms after the stimulus presentation, which suggests that the type of semantics captured by the Statistical model is not present, to a large extent, before 290 ms. We interpret the findings in light of the complementary model that expresses the level of abstractness, focusing on the brain areas revealed by both the Statistical model RSA and the Abstractness model RSA.

The overlap discovered between the Statistical model RSA and Abstractness model RSA is in line with the common finding that processing of abstract words (nouns in particular) activates classical language areas more strongly than processing of concrete words (see Figure S5, for a meta‐analysis, see Wang et al., 2010). Given the size of the searchlight patches used for the RSA analysis (2 cm), only a rough analysis of the cortical areas involved can be made. Among the classical language areas, we observed effects in the inferior frontal gyrus and the middle/superior temporal gyrus. The left inferior frontal gyrus, in particular, has been highlighted as an especially informative area in classification of the abstract/concrete word class (Wang, Baucom, & Shinkareva, 2013). In the present study, overlap between the two RSA maps was observed in the left frontal cortex (inferior areas 410–450 ms; superior areas 320–370 ms) and temporal cortex (320–430 ms). In previous studies, more activity for abstract than for concrete words in these areas was interpreted to reflect greater engagement of the verbal system for processing of abstract concepts (Wang et al., 2010). It is therefore not surprising that the information processed in these classical language areas (i.e., inferior frontal cortex and posterior temporal cortex) mirrors both the statistically derived categorical structure and especially the questionnaire‐based abstract‐concrete dimension. The present RSA findings further suggest that the adjacent superior and medial parts of the frontal cortex are also able to capture the abstractness dimension of word meanings.

The present RSA results also identified areas that in previous studies have shown stronger activation to concrete than abstract words (Wang et al., 2010) or have been robustly linked to object‐specific semantics of concrete objects (Clarke & Tyler, 2014), namely the posterior cingulate, precuneus, fusiform gyrus, and parahippocampal gyrus. In previous studies, increased activation for concrete words has often been interpreted in terms of grounding conceptual information to the perceptual system, particularly in the ventral or dorsal visual processing streams (Binder, Westbury, McKiernan, Possing, & Medler, 2005; Wang et al., 2010). The present findings show that the patterns of activation in these areas are also correlated with the Statistical model derived from corpus data.

The Statistical model RSA and Abstractness model RSA both revealed semantic encoding in the lateral and medial parts of the anterior temporal lobe at 320–430 ms (including ventro‐medial regions). The anterior temporal cortex is well‐known for its role in both semantic dementia (Patterson, Nestor, & Rogers, 2007) and associative semantics (Price, 2012). In light of the associative nature of the statistical semantic model, the present results support the notion that this region is in some manner also linked with processing of word meanings through their associative properties to other words.

A prominent overlap between the Statistical model RSA and Abstractness model RSA was additionally observed in the left precentral cortex. This region has previously been linked to category‐specific semantic activation related to body parts and shape (Pulvermuller, Kherif, Hauk, Mohr, & Nimmo‐Smith, 2009). Here we show that the activity pattern in this region also aligns with the abstract‐concrete structure in the Statistical model.

It is worth noting that the temporal dimension of the decoding and the main RSA findings broadly align with the typical sustained response to visual words at about 400 ms after stimulus onset (the so‐called N400/N400m; see the evoked responses in the Supplementary information). Abstractness model RSA results seem to suggest that processing related only to the abstractness dimension lasts a little longer (especially in the anterior temporal lobe) than the semantic processing captured by the Statistical model. This result may be related to the small difference seen in the temporal dynamics of the evoked responses in some of the channels over the left temporal cortex.

Self‐organization of the semantic space provides an account of how differences along the abstract‐concrete dimension could arise. If the premise stands that neural representations of word meanings arise from similar computational principles as the ones that govern the Statistical model, then words that co‐occur in the environment would also share some aspects of their neural representation (Li & Zhao, 2013). This could lead to categorical groupings that may give rise to the type of categorical differences observed in previous experimental and clinical studies (Wang et al., 2010).

Most of the cortical areas discovered in the RSA analyses align with classical language areas, outside of the primary motor or sensory areas. This suggests that the abstractness dimension is more than a mere reflection of direct sensory‐motor associations, put forward by some advocates of the embodied cognition view (Binder et al., 2016; Kiefer & Pulvermüller, 2012). This finding would explain why previous attempts at decoding abstract words based on sensory‐motor attributes have been unsuccessful (Fernandino et al., 2015) whereas even a crude nominal categorical classification of the abstractness dimension seems to work (Wang et al., 2013). When using a more detailed description of the semantic space, such as the present corpus‐derived Statistical model, we were able to decode MEG signals of individually presented written words; the written modality has previously proven to be challenging even in categorical classification of concrete words (Simanova et al., 2014).

In all neuroimaging research, the choice of task plays a crucial role in terms of how the brain is activated and how the activity can be interpreted. Here, the participants were asked, during catch trials that followed the stimulus word, to evaluate whether the previously presented word would make sense as the first word of the presented sentence. This task cannot be done on superficial linguistic information alone, which may be a weakness of tasks such as lexical decision or word association (Barsalou, Santos, Simmons, & Wilson, 2008). However, the task does include a working memory component, in that the stimulus words must be kept active until the participant knows if a catch trial will appear. This is admittedly different from how meanings are processed in real life. Moreover, we cannot exclude the possibility that the conceptual knowledge may only have been accessed post hoc when needed in a catch trial. However, these possible caveats would impact the Statistical model RSA and the Abstractness model RSA in the same manner, and as the semantic models are qualitatively different, comparing their RSA results is still informative.

The present results suggest that the choice of semantic model used to describe the semantic space does indeed matter. Despite the marked overlap between the Statistical model RSA and the Abstractness model RSA, several areas were uniquely highlighted by only one of the models. This suggests that the Statistical model does not capture all aspects of the abstract‐concrete dimension (or these aspects may remain below the statistical significance threshold). Similarly, while the word2vec model is a well‐argued model of distributional semantics, alternative models such as those based on behavioral feature descriptions may provide complementary views to the semantic system.

On a theoretical level, the human equivalent of the statistical model could be thought of as deriving word meanings from their statistical properties within language, thus essentially grounding the meaning of words to other words. In order for this process to start, a person needs to have at least some vocabulary in place derived by, for example, motor and sensory pairings. Nonetheless, the meanings of these words may also be refined as the vocabulary grows, though whether a word can ever be solely grounded in language without at least a partial sensory‐motor association remains an open question. Moreover, different sources of learning data may also load differently to different regions in the brain, as it has been shown that reading induced fMRI signals in the posterior‐parietal/lateral‐temporal/inferior‐frontal region shows the strongest correlation with text‐models, whereas activity in ventral‐temporal and lateral‐occipital regions shows stronger correlation with image‐based semantic models (Anderson, Bruni, Lopopolo, Poesio, & Baroni, 2015).

The notion of grounding words to language should be seen as an attempt to reconcile the perhaps artificial disparities between the distributed and embodied views of semantics (Andrews et al., 2014). It alludes to the fact that the computational principles that govern the brain such as Hebbian learning (Hebb, 1949) and basic principles of optimization (Friston, 2012; Zipf, 1949) can take advantage of any environment, be it physical or linguistic. Future research may thus need to focus not only on the type of data that is used but also on the process and computational principles by which the words become represented in the brain. In our view, emergent categorical structures pose a tempting computational solution to how the organization of the semantic space may arise.

## 5 CONCLUSION

The present study used a computationally explicit framework to evaluate how semantic representations can be expressed in the brain as a result of statistical regularities in our language environment and computational principles known to exist in the brain. We were able to link specific cortical areas to semantic representations, describe the type of information that could be processed there, and demonstrate that this information can arise merely through statistical co‐occurrences in the language environment. We show that a statistical model is sufficient to account for a substantial part (i.e., enough to enable successful encoding) of the semantic processing. This may be taken as proof of concept that exposure to language in itself can serve a similar purpose as other sensory, motor, emotional or perceptual experiences in forming neural representations of word meanings. In theory, this could mean that abstract words, in particular, could be grounded in the language experience, thereby overcoming the need for physical referent.

## Supporting information

**Supplementary Figure S1** Cosine distance between the semantic location predicted from the MEG data and the semantic location indicated by the word2vec model, averaged across items. The 99% confidence interval reflects the variation among different individuals. The blue line indicates the *p* < .05 significance threshold as determined by a permutation test (1,000 permutations, performed for each time point separately). CI = confidence interval.
**Supplementary Figure S2**: Decoding prediction accuracy for each stimulus‐item pair, averaged across participants.
**Supplementary Figure S3**: Within‐category decoding. The significance threshold for the time resolved decoding was determined to be 63% through a permutation test.
**Supplementary Figure S4**: Grand average evoked fields comparing the gradiometer time‐courses of abstract words versus those of concrete words. Shown is the vector magnitude of the signal at each gradiometer pair. The insets show a magnification of the signal at two sensor locations.
**Supplementary Figure S5**: Minimum‐norm (dSPM) source estimates for the response to concrete words (A) and abstract words (B), and the difference between the two (C), averaged across stimuli and participants. Shown are the average MNE‐dSPM values for time windows of 60 ms, in the left hemisphere.
**Supplementary Figure S6**: Representational similarity analysis (RSA) using partial Spearman correlation showing the unique contribution of each semantic model in modeling the brain data. If an area is highlighted by both models, it implies that each model explains different aspects of the data in the same region.
**Supplementary Table S1**. Full list of stimuli

## Data Availability

The text corpus containing 1.5 billion Finnish words used to derive the statistical model cannot be publicly distributed due to the Finnish copyright law limitations. It is available upon request for research purposes, for contact information see http://bionlp.utu.fi/finnish-internet-parsebank.html. The word2vec models used in this study (derived from the abovementioned corpus), together with the custom code used in the study can be accessed at https://github.com/AaltoImagingLanguage/hulten2021. The code to compute RSA can be found at https://github.com/wmvanvliet/mne-rsa. The stimulus words are publicly available and listed in Table S1. The MEG and MRI data are available upon reasonable request from the authors; the data is not publicly available as it contains personal information, and its reuse for other research purposes requires renewed consent from the participants and a new ethical pre‐review.
