# Supplementary material for: The neural representation of abstract words may arise through grounding word meaning in language itself
Source: Hum Brain Mapp. 2021 Jul 15;42(15):4973–84. doi: 10.1002/hbm.25593 (PMC8449102; doi:10.1002/hbm.25593)
Supplement: Supplementary file 1 — Supplementary Figure S1 Cosine distance between the semantic location predicted from the MEG data and the semantic location indicated by the word2vec model, averaged across items. The 99% confidence interval reflects the variation among different individuals. The blue line indicates the p < .05 significance threshold as determined by a permutation test (1,000 permutations, performed for each time point separately). CI = confidence interval. Supplementary Figure S2: Decoding prediction accuracy for each stimulus‐item pair, averaged across participants. Supplementary Figure S3: Within‐category decoding. The significance threshold for the time resolved decoding was determined to be 63% through a permutation test. Supplementary Figure S4: Grand average evoked fields comparing the gradiometer time‐courses of abstract words versus those of concrete words. Shown is the vector magnitude of the signal at each gradiometer pair. The insets show a magnification of the signal at two sensor locations. Supplementary Figure S5: Minimum‐norm (dSPM) source estimates for the response to concrete words (A) and abstract words (B), and the difference between the two (C), averaged across stimuli and participants. Shown are the average MNE‐dSPM values for time windows of 60 ms, in the left hemisphere. Supplementary Figure S6: Representational similarity analysis (RSA) using partial Spearman correlation showing the unique contribution of each semantic model in modeling the brain data. If an area is highlighted by both models, it implies that each model explains different aspects of the data in the same region. Supplementary Table S1. Full list of stimuli [file HBM-42-4973-s001.docx]

# Supplementary Material

## Cosine distance over time between the model prediction and the ground truth

To get a sense of how "close" the zero-shot decoding model came to predicting the correct location of each item in the semantic space, we computed the cosine distance between the semantic location predicted from the MEG data and the semantic location indicated by the word2vec model.


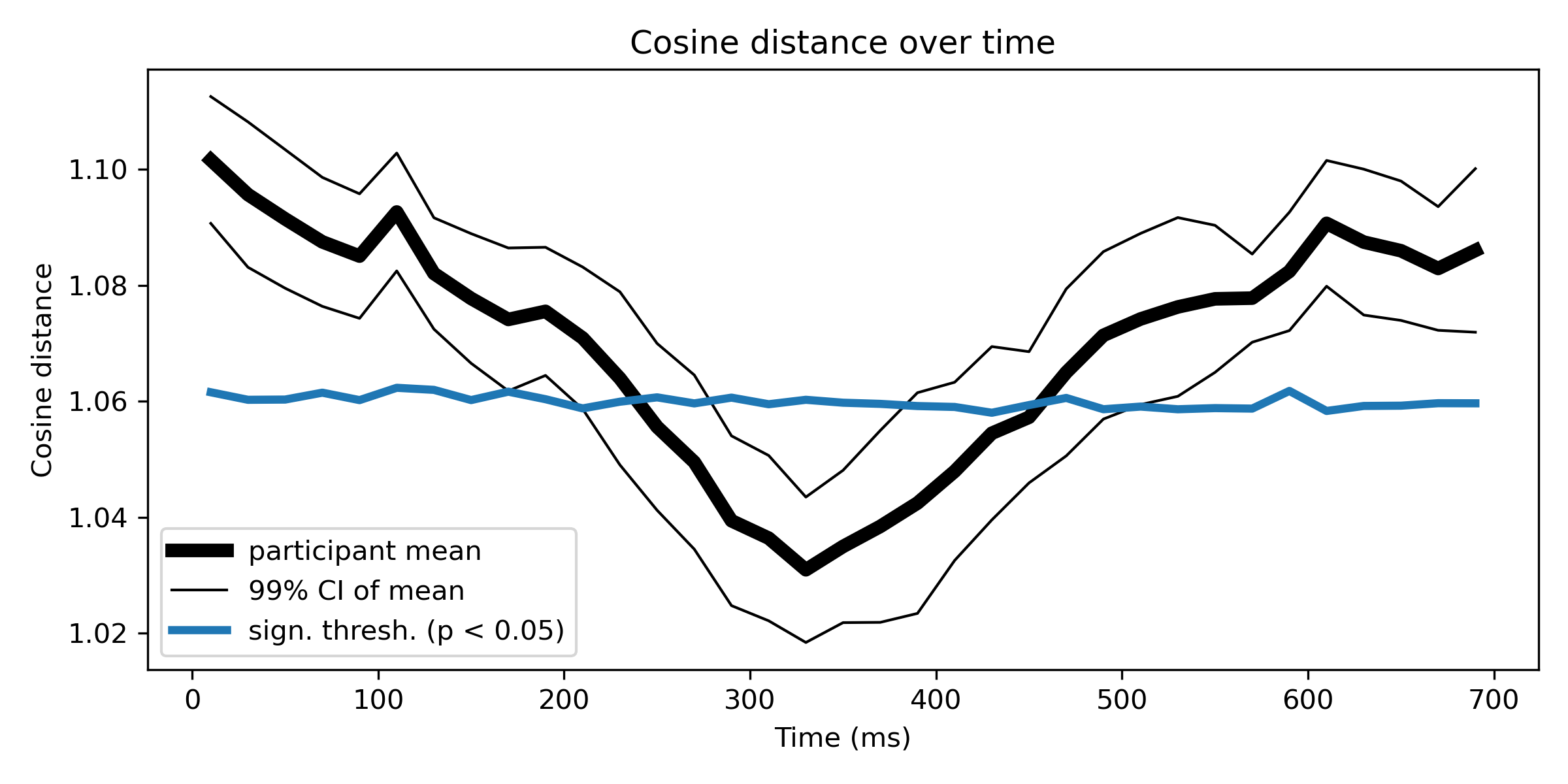


## *Supplementary Figure 1:* Cosine distance between the semantic location predicted from the MEG data and the semantic location indicated by the word2vec model, averaged across items. The 99 % confidence interval reflects the variation among different individuals. The blue line indicates the p < 0.05 significance threshold as determined by a permutation test (1000 permutations, performed for each time point separately). CI = confidence interval.

##

## Breakdown of decoding accuracy

In order to understand if any underlying systematics in the stimulus selection was driving the performance of the zero-shot decoding model, we looked at the pairwise comparisons in the leave-two-out cross validation scheme. Figure S1 shows the performance of each combination of item-pairs averaged across all participants. As no clear categorical pattern emerges, we conclude that the prediction accuracy is unlikely to be solely driven by a specific category of the stimulus words.


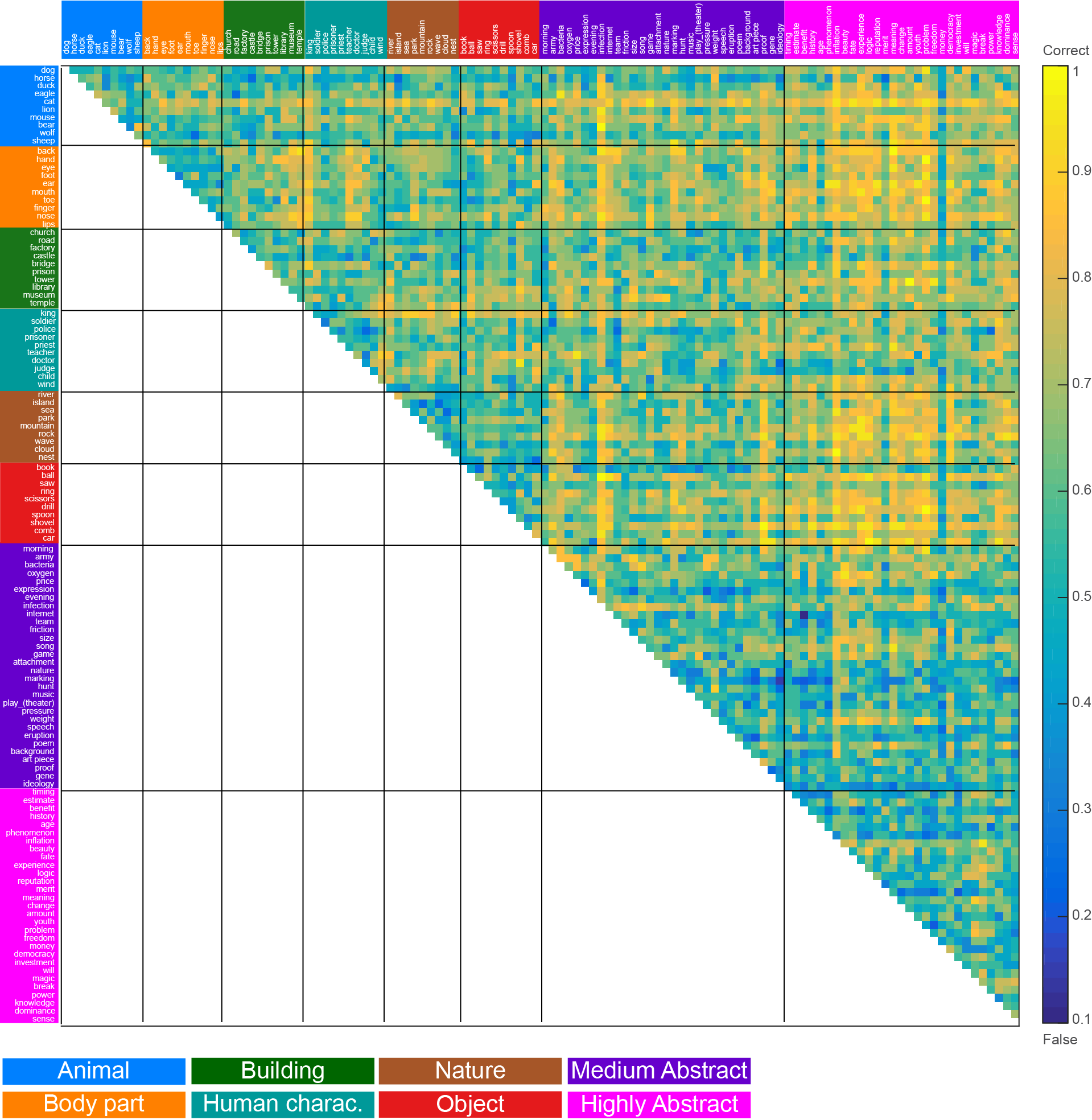


*Supplementary Figure 2:* Decoding prediction accuracy for each stimulus-item pair, averaged across participants*.*

##

## Decoding performance, using only within-category item pairs


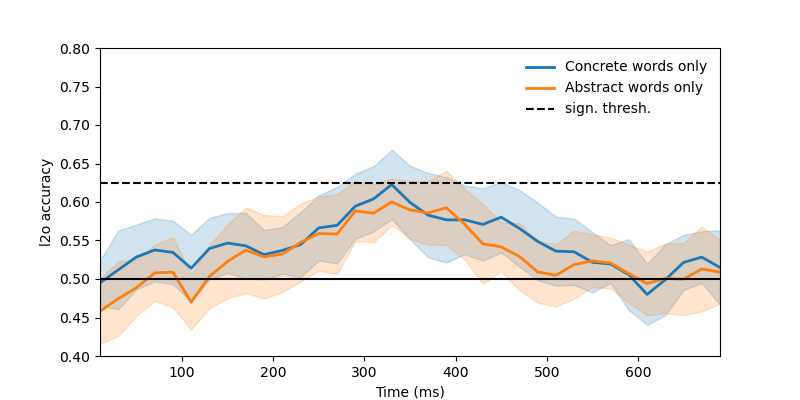


## *Supplementary Figure 3:* Within-category decoding. The significance threshold for the time resolved decoding was determined to be 63% through a permutation test.

##

## Grand average evoked fields


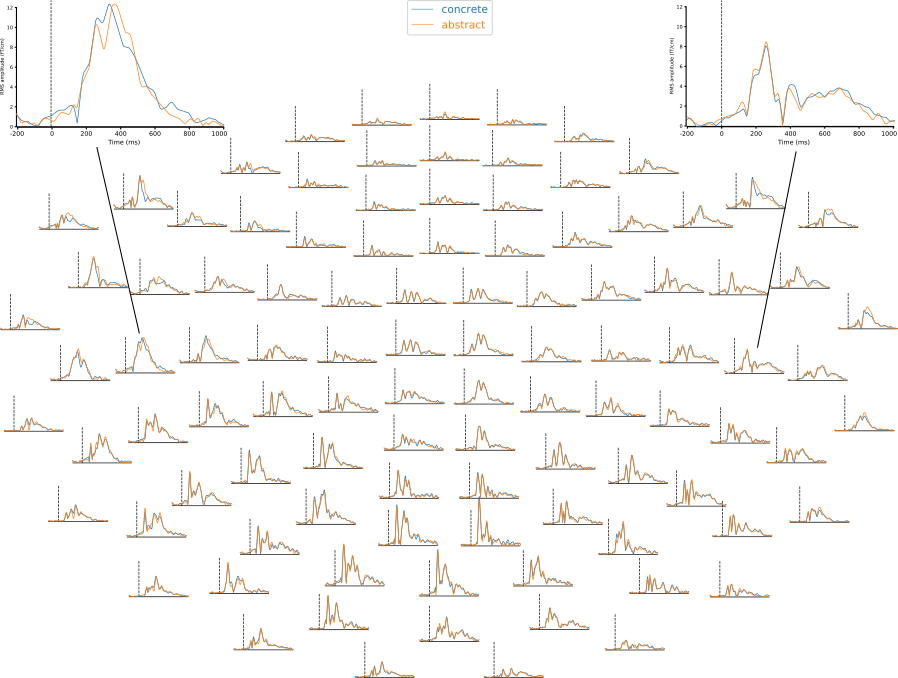
*Supplementary Figure 4:* Grand average evoked fields comparing the gradiometer time-courses of abstract words versus those of concrete words. Shown is the vector magnitude of the signal at each gradiometer pair. The insets show a magnification of the signal at two sensor locations.

## Minimum norm estimate (MNE) of source activation


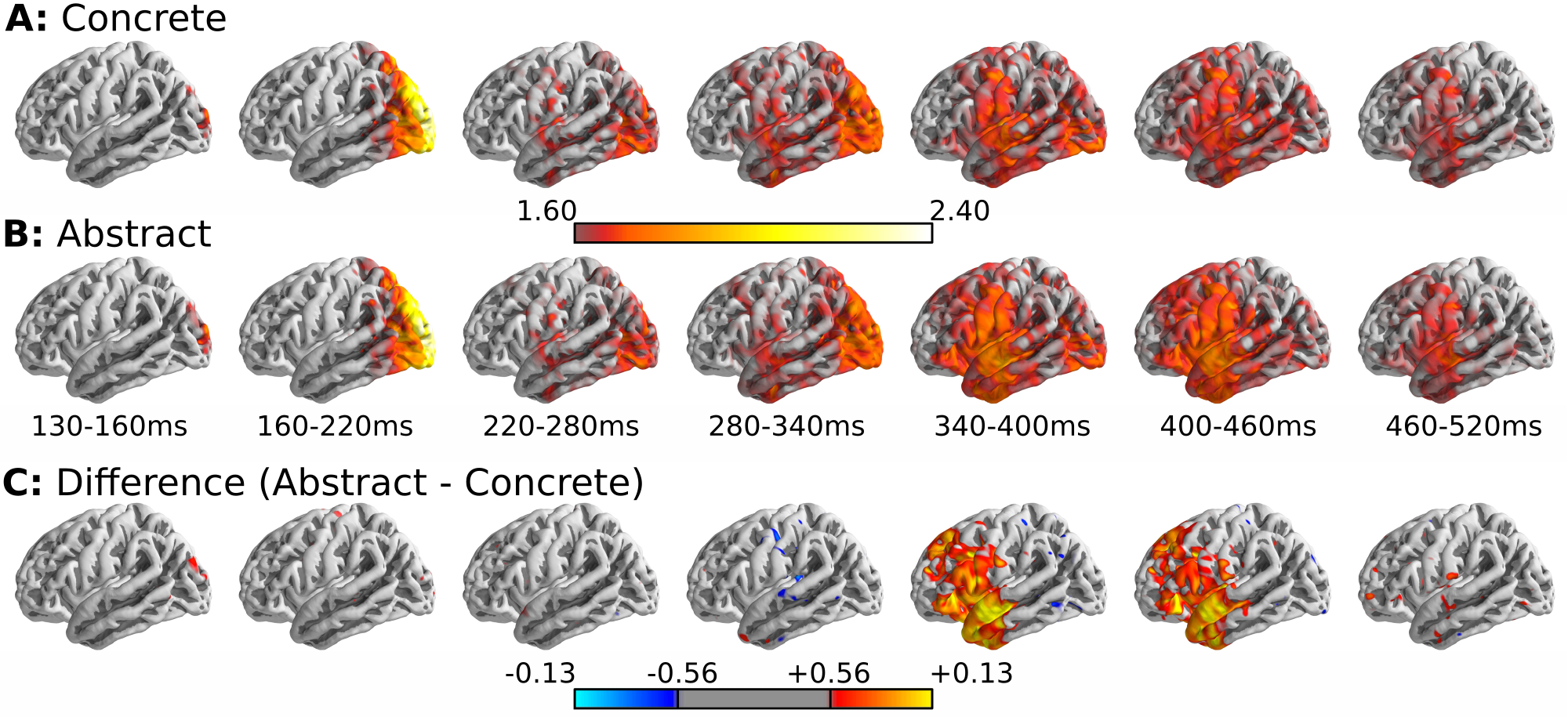


*Supplementary Figure 5:* Minimum-norm (dSPM) source estimates for the response to concrete words (A) and abstract words (B), and the difference between the two (C), averaged across stimuli and participants. Shown are the average MNE-dSPM values for time windows of 60 ms, in the left hemisphere.

## RSA analysis using Partial correlation


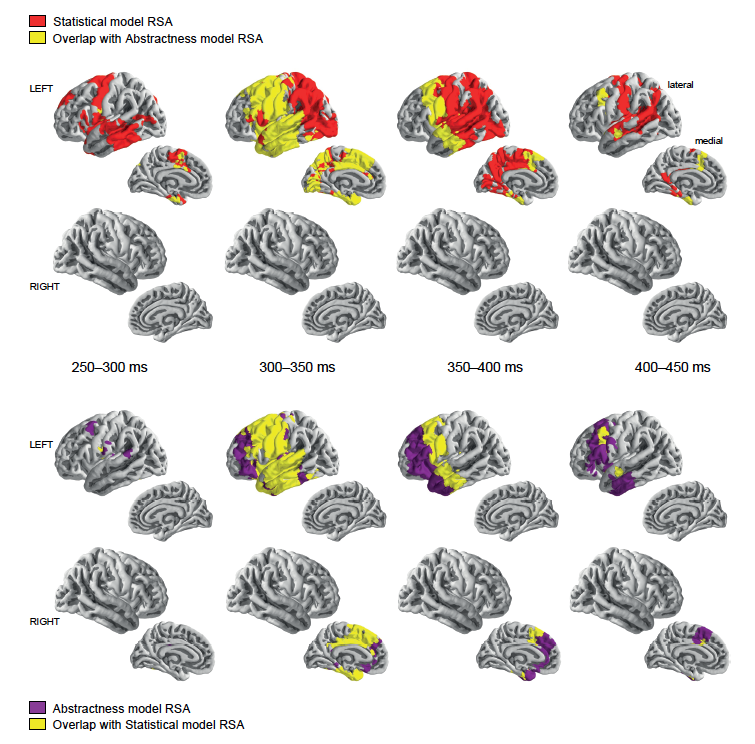


*Supplementary figure 6: Representational similarity analysis (RSA) using partial Spearman correlation showing the unique contribution of each semantic model in modeling the brain data. If an area is highlighted by both models, it implies that each model explains different aspects of the data in the same region.*

## Supplementary Table 1. Full list of stimuli

| Stimulus | Translation | Category | Length | Lemma Frequency | Bigramfreq_sum |
| --- | --- | --- | --- | --- | --- |
| koira | dog | Animal | 5.00 | 685356.00 | 167870177.00 |
| hevonen | horse | Animal | 7.00 | 235379.00 | 282477964.00 |
| ankka | duck | Animal | 5.00 | 26259.00 | 227191282.00 |
| kotka | eagle | Animal | 5.00 | 8965.00 | 204227072.00 |
| kissa | cat | Animal | 5.00 | 157935.00 | 198955045.00 |
| leijona | lion | Animal | 7.00 | 51118.00 | 170386956.00 |
| hiiri | mouse | Animal | 5.00 | 71638.00 | 112786105.00 |
| karhu | bear | Animal | 5.00 | 37820.00 | 161508406.00 |
| susi | wolf | Animal | 4.00 | 49407.00 | 186210548.00 |
| lammas | sheep | Animal | 6.00 | 44329.00 | 271537051.00 |
|  | | **Category mean** | 5.40 | 136820.60 | 198315060.60 |
|  |  | **sd** | 0.97 | 204725.36 | 51464521.83 |
| selkä | back | Body part | 5.00 | 141898.00 | 177927894.00 |
| käsi | hand | Body part | 5.00 | 111515.00 | 153390074.00 |
| silmä | eye | Body part | 5.00 | 378313.00 | 172062724.00 |
| jalka | foot | Body part | 5.00 | 288091.00 | 267654785.00 |
| korva | ear | Body part | 4.00 | 625010.00 | 129653660.00 |
| suu | mouth | Body part | 3.00 | 177382.00 | 73257394.00 |
| varvas | toe | Body part | 6.00 | 23690.00 | 236862114.00 |
| sormi | finger | Body part | 5.00 | 78317.00 | 92424633.00 |
| nenä | nose | Body part | 4.00 | 56284.00 | 228755091.00 |
| huulet | lips | Body part | 6.00 | 38179.00 | 148597926.00 |
|  | | **Category mean** | 4.80 | 191867.90 | 168058629.50 |
|  |  | **sd** | 0.92 | 189729.94 | 62563223.25 |
| kirkko | church | Building | 6.00 | 346349.00 | 198755314.00 |
| tie | road | Building | 3.00 | 386080.00 | 106539169.00 |
| tehdas | factory | Building | 6.00 | 90380.00 | 177837616.00 |
| linna | castle | Building | 5.00 | 79070.00 | 282044623.00 |
| silta | bridge | Building | 5.00 | 29077.00 | 309732105.00 |
| vankila | prison | Building | 7.00 | 45905.00 | 384391355.00 |
| torni | tower | Building | 5.00 | 35038.00 | 111906624.00 |
| kirjasto | library | Building | 8.00 | 169639.00 | 375900974.00 |
| museo | museum | Building | 5.00 | 115741.00 | 159874013.00 |
| temppeli | temple | Building | 8.00 | 33462.00 | 275203142.00 |
|  | | **Category mean** | 5.80 | 133074.10 | 238218493.50 |
|  |  | **sd** | 1.55 | 130733.96 | 101850077.60 |
| kuningas | king | Human character | 8.00 | 89374.00 | 327803527.00 |
| sotilas | soldier | Human character | 7.00 | 57594.00 | 341991067.00 |
| poliisi | police | Human character | 7.00 | 301478.00 | 378437306.00 |
| vanki | prisoner | Human character | 5.00 | 43619.00 | 211497872.00 |
| pappi | priest | Human character | 5.00 | 60550.00 | 94369458.00 |
| opettaja | teacher | Human character | 8.00 | 246973.00 | 443120060.00 |
| lääkäri | doctor | Human character | 7.00 | 181418.00 | 180835905.00 |
| tuomari | judge | Human character | 7.00 | 113697.00 | 288789868.00 |
| lapsi | child | Human character | 5.00 | 1418872.00 | 238047831.00 |
|  | | **Category mean** | 6.05 | 264430.90 | 260674297.16 |
|  |  | **sd** | 1.97 | 414413.91 | 116070236.10 |
| tuli | wind | Nature | 4.00 | 51894.00 | 175870708.00 |
| joki | river | Nature | 4.00 | 239509.00 | 139214957.00 |
| saari | island | Nature | 5.00 | 161834.00 | 233855559.00 |
| meri | sea | Nature | 4.00 | 129534.00 | 120037265.00 |
| puisto | park | Nature | 6.00 | 74059.00 | 259921668.00 |
| vuori | mountain | Nature | 5.00 | 67885.00 | 125812300.00 |
| kallio | rock | Nature | 6.00 | 43488.00 | 392168880.00 |
| aalto | wave | Nature | 5.00 | 35885.00 | 241692312.00 |
| pilvi | cloud | Nature | 5.00 | 52215.00 | 108115386.00 |
| pesä | nest | Nature | 4.00 | 37354.00 | 73918027.00 |
|  | | **Category mean** | 4.80 | 89365.70 | 187060706.20 |
|  |  | **sd** | 0.79 | 67130.34 | 95594062.73 |
| kirja | book | Object | 5.00 | 757126.00 | 175682376.00 |
| pallo | ball | Object | 5.00 | 131644.00 | 262781831.00 |
| saha | saw | Object | 4.00 | 18615.00 | 84491401.00 |
| sormus | ring | Object | 6.00 | 25194.00 | 137328551.00 |
| sakset | scissors | Object | 6.00 | 7992.00 | 191391516.00 |
| pora | drill | Object | 4.00 | 7755.00 | 65614066.00 |
| lusikka | spoon | Object | 7.00 | 14151.00 | 397668837.00 |
| lapio | shovel | Object | 5.00 | 6623.00 | 185073292.00 |
| kampa | comb | Object | 5.00 | 4983.00 | 166477942.00 |
| auto | car | Object | 4.00 | 689136.00 | 131121750.00 |
|  | | **Category mean** | 5.10 | 166321.90 | 179763156.20 |
|  |  | **sd** | 0.99 | 296314.93 | 94871232.21 |
| aamu | morning | Medium Abstract | 4.00 | 379139.00 | 145104158.00 |
| armeija | army | Medium Abstract | 7.00 | 79931.00 | 199362973.00 |
| bakteeri | bacteria | Medium Abstract | 8.00 | 27189.00 | 213518970.00 |
| happi | oxygen | Medium Abstract | 5.00 | 25132.00 | 81501535.00 |
| hinta | price | Medium Abstract | 5.00 | 1126169.00 | 382993097.00 |
| ilme | expression | Medium Abstract | 4.00 | 74099.00 | 82498688.00 |
| ilta | evening | Medium Abstract | 4.00 | 497767.00 | 221870966.00 |
| infektio | infection | Medium Abstract | 8.00 | 10631.00 | 256833422.00 |
| internet | internet | Medium Abstract | 8.00 | 119777.00 | 451022648.00 |
| joukkue | team | Medium Abstract | 7.00 | 285916.00 | 181129566.00 |
| kitka | friction | Medium Abstract | 5.00 | 6469.00 | 261947511.00 |
| koko | size | Medium Abstract | 4.00 | 1595650.00 | 146049269.00 |
| laulu | song | Medium Abstract | 5.00 | 152433.00 | 225977302.00 |
| leikki | game | Medium Abstract | 6.00 | 52374.00 | 229247037.00 |
| liite | attachment | Medium Abstract | 5.00 | 49527.00 | 266540989.00 |
| luonto | nature | Medium Abstract | 6.00 | 226253.00 | 223600811.00 |
| merkintä | marking | Medium Abstract | 8.00 | 68864.00 | 438072123.00 |
| metsästys | hunt | Medium Abstract | 9.00 | 29285.00 | 246030754.00 |
| musiikki | music | Medium Abstract | 8.00 | 409352.00 | 389644253.00 |
| näytelmä | play_(theater) | Medium Abstract | 8.00 | 54204.00 | 247713896.00 |
| paine | pressure | Medium Abstract | 5.00 | 68437.00 | 361583346.00 |
| paino | weight | Medium Abstract | 5.00 | 140988.00 | 296297519.00 |
| puhe | speech | Medium Abstract | 4.00 | 174018.00 | 51475696.00 |
| purkaus | eruption | Medium Abstract | 7.00 | 7625.00 | 260532941.00 |
| runo | poem | Medium Abstract | 4.00 | 111417.00 | 56234579.00 |
| tausta | background | Medium Abstract | 6.00 | 186679.00 | 520805403.00 |
| teos | artwork | Medium Abstract | 4.00 | 280865.00 | 117047913.00 |
| todiste | proof | Medium Abstract | 7.00 | 47511.00 | 314244783.00 |
| geeni | gene | Medium Abstract | 5.00 | 26817.00 | 171703006.00 |
|  | | **Category mean** | 5.90 | 217742.00 | 242778798.41 |
|  |  | **sd** | 1.61 | 347449.54 | 118662770.43 |
| aate | ideology | Highly Abstract | 4.00 | 22418.00 | 218254904.00 |
| ajoitus | timing | Highly Abstract | 7.00 | 12713.00 | 338155259.00 |
| arvio | estimate | Highly Abstract | 5.00 | 167420.00 | 97911242.00 |
| etu | benefit | Highly Abstract | 3.00 | 242585.00 | 112175587.00 |
| historia | history | Highly Abstract | 8.00 | 302404.00 | 327749129.00 |
| ikä | age | Highly Abstract | 3.00 | 233810.00 | 89370757.00 |
| ilmiö | phenomenon | Highly Abstract | 5.00 | 124740.00 | 115026613.00 |
| inflaatio | inflation | Highly Abstract | 9.00 | 18545.00 | 517134066.00 |
| kauneus | beauty | Highly Abstract | 7.00 | 50494.00 | 325046028.00 |
| kohtalo | fate | Highly Abstract | 7.00 | 59201.00 | 354499549.00 |
| kokemus | experience | Highly Abstract | 7.00 | 460564.00 | 251847716.00 |
| logiikka | logic | Highly Abstract | 8.00 | 31718.00 | 272576494.00 |
| maine | reputation | Highly Abstract | 5.00 | 60427.00 | 382834756.00 |
| meriitti | merit | Highly Abstract | 8.00 | 5412.00 | 418266918.00 |
| merkitys | meaning | Highly Abstract | 8.00 | 288413.00 | 288028643.00 |
| muutos | change | Highly Abstract | 6.00 | 462729.00 | 218865836.00 |
| määrä | amount | Highly Abstract | 5.00 | 715303.00 | 100907805.00 |
| nuoruus | youth | Highly Abstract | 7.00 | 32593.00 | 193989767.00 |
| ongelma | problem | Highly Abstract | 7.00 | 692366.00 | 186862917.00 |
| vapaus | freedom | Highly Abstract | 6.00 | 99771.00 | 218295721.00 |
| raha | money | Highly Abstract | 4.00 | 549237.00 | 73619031.00 |
| demokratia | democracy | Highly Abstract | 10.00 | 77215.00 | 252431804.00 |
| sijoitus | investment | Highly Abstract | 8.00 | 92187.00 | 418842948.00 |
| tahto | will | Highly Abstract | 5.00 | 91480.00 | 268246391.00 |
| taika | magic | Highly Abstract | 5.00 | 18932.00 | 417527683.00 |
| tauko | break | Highly Abstract | 5.00 | 90381.00 | 272764335.00 |
| teho | power | Highly Abstract | 4.00 | 82320.00 | 99392184.00 |
| tieto | knowledge | Highly Abstract | 5.00 | 1622531.00 | 209499704.00 |
| valta | dominance | Highly Abstract | 5.00 | 196627.00 | 315318012.00 |
| järki | sense | Highly Abstract | 5.00 | 87908.00 | 112104088.00 |
|  | | **Category mean** | 6.03 | 233081.47 | 248918196.23 |
|  |  | **sd** | 1.77 | 330138.35 | 118064526.25 |
